# Beta Blockers Up-Titration in Patients with Heart Failure Reduced Ejection Fraction and Cardiac Resynchronization Therapy, a Single Center Study

**DOI:** 10.3390/medsci7060071

**Published:** 2019-06-18

**Authors:** Daniele Masarone, Marina Verrengia, Ernesto Ammendola, Rita Gravino, Fabio Valente, Rossella Vastarella, Marta Rubino, Giuseppe Limongelli, Giuseppe Pacileo

**Affiliations:** 1 Heart Failure Unit, AORN Colli, 80121 Naples, Italy; mariverr@yahoo.it (M.V.); ammendolaernesto@libero.it (E.A.); ritagravino@virgilio.it (R.G.); fabioval85@gmail.com (F.V.); rossellavastarella86@gmail.com (R.V.); rubinomarta@libero.it (M.R.); limongelligiuseppe@libero.it (G.L.); gpacileo58@gmail.com (G.P.); 2 Department of Translational Medical Sciences, Luigi Vanvitelli University, 80121 Naples, Italy; 3 Institute of Cardiovascular Sciences, University College of London, London WC1E 6BT, UK

**Keywords:** heart failure reduced ejection fraction, beta-blockers, cardiac resynchronization therapy

## Abstract

Clinical trials have shown the benefits of β-blockers therapy in patients with heart failure reduced ejection fraction. These benefits include improved survival and a reduced need for hospitalization. Cardiac resynchronization therapy has emerged as an essential device-based therapy for symptomatic patients with heart failure reduced ejection fraction despite optimal pharmacologic treatment. The extent to which β-blockers are being utilized in patients receiving cardiac resynchronization therapy is not well known. In this study, we evaluate the possibility of increasing β-blockers doses in an unselected cohort of heart failure reduced ejection patients after cardiac resynchronization therapy capable defibrillator system implantation and the correlation between β-blockers treatments and clinical outcome. Methods and results: Patients with heart failure reduced ejection fraction in β-blockers therapy that underwent cardiac resynchronization therapy capable defibrillator system implantation between July 2008, and December 2016 were enrolled in the study. The β-blockers dose was determined at the time of discharge and during follow-up. Cardiovascular mortality, hospitalization for worsening heart failure or arrhythmic storm and appropriate intervention of the device, were recorded. The study cohort included 480 patients, 289 patients (60.3%) had β-blockers doses equal to the dose before CRT (Group 1), 191 patients (39.7%) had higher β-blockers doses than those before the CRT implant (Group 2). Comparing the two groups, Group 2 have lower cardiovascular mortality, heart failure-related hospitalization, and arrhythmic events than Group 1. Conclusion: After initiating CRT, β-blockers could be safely up-titrated at higher doses with the reduction in mortality, heart failure-related hospitalization, and arrhythmic events.

## 1. Introduction

Traditional teaching in 1980s was that β-blockers (BBs) should be avoided in patients with congestive heart failure (HF) [1]. The basis of this hypothesis was that the sympathetic nervous system provided support of the failing heart, so attenuating sympathetic system activity with BBs would precipitate or exacerbate HF [2]; nevertheless, pathophysiological evidence collected in the past decades has documented that neurohormonal hyperactivation has a crucial role in the progression of HF syndrome [3,4]. As consequence of this new pathophysiological view, the use and the efficacy of BBs in the treatment of HF reduced ejection fraction (HFrEF) has been investigated in numerous randomized clinical trials [5,6,7] (Table 1) that provide a rational basis for their inclusion as the cornerstone of therapy in international guidelines [8,9].

More recently ventricular dyssynchrony has been recognized as an important therapeutic target in patients with HFrEF [10,11], in up to 30% of patients with HFrEF an interventricular conduction delay (QRS duration ≥ 120 ms) is present and associated with a delayed onset of left ventricle systole, decreased systolic function, and worsened survival [12]. Therapeutic options targeted at restoring normal mechanical synchrony, such as cardiac resynchronization therapy (CRT) [13], have been shown to enhance myocardial contractility without increasing myocardial oxygen consumption and also improved survival in symptomatic patients with HFrEF and QRS duration > 120–150 ms [14,15]. In addition, after CRT BBs can be introduced and up-titrated in HF patients [16].

In this study, we evaluate the effect of CRT on the up-titration of β-blockers doses and the correlation between high BBs doses and clinical outcome.

## 2. Materials and Methods

Patients enrollment was started in July 2008 and completed in December 2016. Out of a total of 550 patients who had undergone CRT implantation, 480 took BBs and were enrolled in the study; the remaining 70 patients were excluded from the study for the presence of asthma (25 patients), or for refusing to sign informed consent (45 patients).

Before CRT implantation, the following baseline patient characteristics were recorded: Patient demographics including age and sex, New York Heart Association (NYHA) functional class, QRS duration, etiology of cardiomyopathy, comorbidities, serum creatinine level, and echocardiographic parameters such as left ventricle end-diastolic volume (LVEDV) and left ventricle ejection fraction (LVEF). After implantation of CRT-D, BBs doses were increased according to our units protocol (doubling of the dose every 4 weeks if clinically possible, up to the maximum tolerated dosage or target dose). The doses of BBs were assessed at the time of discharge and at one year after CRT implantation; according to the latter, patients were divided into Group 1 (patients with BBs doses equal to the dose before CRT) and Group 2 (patients with higher BBs doses than those with previous CRT implant). Cardiovascular mortality, hospitalization for worsening HF or arrhythmic storm and appropriate shock of CRT-D were recorded in both groups.

The study was conducted according to the declaration of Helsinki and approved by the local ethics committee (deliberation n° 438; June 2008). All patients signed a consent form.

## 3. Statistical Analysis

Clinical characteristics were presented in tabular form for the overall population and subgroups defined by BBs dose. Efficacy and safety outcomes during follow-up were summarized using standard descriptive statistics. The Mann-Whitney U test was used to compare outcomes across BBs doses.

All statistical analyses were performed using SPSS 25.0 (IBM, Armonk, NY, USA).

All *p* values were two-sided, and *p* < 0.05 was considered to be statistically significant.

## 4. Results

### 4.1. Baseline Characteristic

The study cohort comprises 480 patients. The baseline demographic and clinical characteristics are shown in Table 2. At enrollment most patients were male (n = 318; 66.2%) with HF due to ischemic cardiomyopathy (n = 270; 56.2%). All patients had severe systolic dysfunction with an average LVEF of 30 ± 4% and prolonged QRS (149 ± 15 ms). There were no significant differences between the two groups of patients’ populations at entry into the study (Table 3).

### 4.2. HF Pharmacotherapy before and after CRT

Before receiving CRT, most patients took inhibitors of the renin-angiotensin-aldosterone system (n 400; 83.3%), while 255 patients (53.1%) assumed a mineralocorticoid antagonist (mainly spironolactone). BBs were used in all patients at the maximum tolerated dose.

After receiving CRT in 289 patients (60.3%), a further increase was not tolerated mainly due to fatigue (defined as a mean value at the fatigue severity scale > 5), on the other hand in 191 (39.7% of patients) a further increase of BBs doses was possible (Table 4).

### 4.3. Clinical and Echocardiographic Response to CRT

In all patients, a percentage of biventricular pacing > 95% was achieved.

Of the overall population, 65% of patients had a clinical or echocardiographic response to CRT, respectively defined as an improvement of the NYHA class or a reduction of at least 15% of LVEDV. The clinical and echocardiographic features of the two groups after CRT are shown in Table 5.

### 4.4. Follow-Up

During follow-up (6.5 + 1.2 years) 55 patients (11.5%) died of cardiovascular causes (35 patients in Group 1 and 20 patients in Group 2). There were 190 admissions for heart failure or arrhythmic storm (122 in Group 1 and 68 in Group 2). Finally, 136 device interventions (i.e., shock by ventricular fibrillation or sustained ventricular tachycardia) occurred during follow-up (90 shocks in Group 1 and 46 shocks in Group 2).

**Figure 1:**
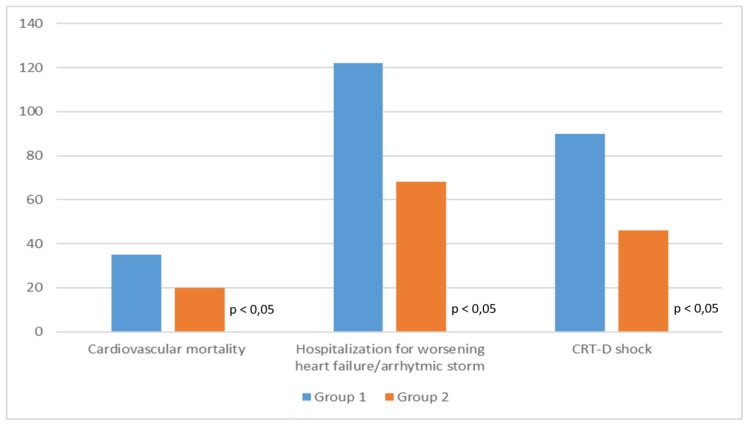
Clinical outcome of the two study groups.

**Figure 2:**
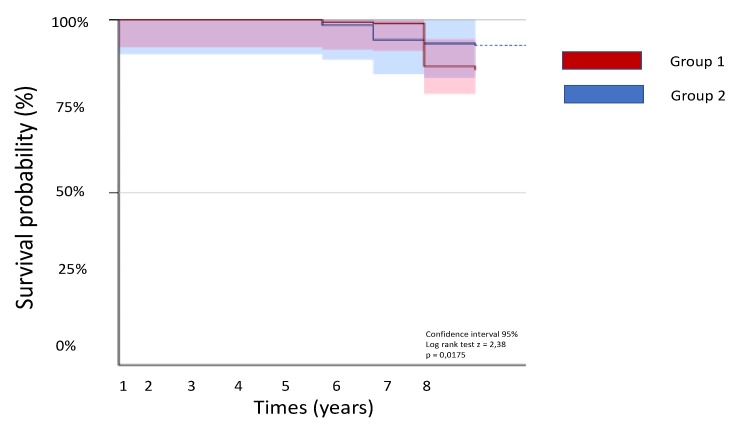
Kaplan-Meier curves of the two study groups.

Comparing the two groups, Group 2 have lower cardiovascular mortality, heart failure-related hospitalization, and arrhythmic events than Group 1 (Figure 1 and Figure 2).

## 5. Discussion

The main results of this study are that, with patients with HFrEF undergoing CRT implantation, it is possible to increase the doses of BBs and that the use of high doses allows better clinical results to be obtained. Initial experience with BBs in HFrEF was reported in 1979 [17]. However, the first randomized multicenter study was published in 1993 [18], while only in 1997 carvedilol was approved for the treatment of HFrEF. The rationale for the slow acceptance of β-blocker therapy in HFrEF is related to the risk of worsening of HF [19]. Nowadays the available data show that carvedilol, bisoprolol, and metoprolol reduce morbidity and mortality in patients with symptomatic HFrEF [20]. However, clinical studies that have demonstrated the efficacy of β-blockers in HFrEF have been conducted before the spread of CRT, and such therapy may attenuate hypotension and symptomatic bradycardia which are significant factors limiting the use of high-dose BBs.

CRT typically increases systolic blood pressure by approximately 5–10 mmHg [21] and setting a low-frequency limit of pacing at 50 b/min allows for titration of BBs at higher doses to achieve a heart rate at rest between 50 and 60 b/min.

For this reason, higher doses of BBs could be used in our population after CRT implantation compared to those commonly used in clinical practice; however, in a percentage of patients, a higher dose was not achieved mainly due to fatigue and asthenia that in our study represent the main factors limiting a high dose of BBs. No other common side effects of BBs (e.g., sexual dysfunction, depression, sleeping difficulties) occurred in our population.

Furthermore, the use of higher doses of BBs is related to better clinical outcome (i.e., reduction in mortality, hospitalization due to heart failure/arrhythmias and implantable cardioverter-defibrillator shock).

While the beneficial effect of BBs is undisputed, it is not yet clear whether the benefit is linked to the achievement of a target heart rate or the achievement of a target dose [22].

Current guidelines recommend up-titration of beta-blockers at the target dose, as established in clinical trials [23]. However, some evidence indicates that the magnitude of heart rate reduction is more important than the same dose [24], in the CIBIS-II study, the decrease in heart rate obtained with bisoprolol was proportionally associated with better survival [25]; even in a meta-analysis comprising more than 17,000 patients with HFrEF on BBs, the extent of heart rate reduction, and not the BBs doses, was related to the reduction in mortality [26]. Recently Fiuzat and collaborators showed in a well-treated HFrEF cohort that the clinical outcome is related to the BBs doses and not to the reduction in heart rate, [27].

In our population, the heart rate at enrollment ranged from 60 to 65 b/min, and no further significant reduction was achieved after the increase in BBs doses, confirming the fact that the clinical benefit is related to the BBs doses and not to the decrease in heart rate.

In addition, no difference in prognosis was found between the BBs, this confirms that all of the BBs approved in international guidelines for the treatment of heart failure have the same impact on clinical outcome.

Finally, because the blood pressure values increase in both the groups of patients the better prognosis is in our opinion given by the increase of the BBs dose and not by a better hemodynamic profile despite the increase of the BBs.

## 6. Conclusions

Our study demonstrated that after initiating CRT, BBs could be safely up-titrated at higher doses with the reduction in cardiovascular mortality, HF-related hospitalization, and arrhythmic events.

## 7. Study Limitation 

The study was retrospective in nature and subject to associated limitations. BBs therapy was not randomly assigned, which raised the potential for indication bias. In addition, the selection of BBs is based on physician choice; therefore, a class effect was assumed and partially demonstrated. Finally, patients with atrial fibrillation were included in the study and, because the presence of atrial fibrillation may affect the positive impact of BBs on clinical outcome, we cannot exclude the possibility that that may have a role in the results.

## Figures and Tables

**Table 1:** Summary of randomized clinical trials of β-blockers in heart failure reduced ejection fraction.

Trial	Year	β-Blockers	n° of Patients	Effects on Mortality
CIBIS	1994	Bisoprolol	641	No significant difference in mortality between the two groups
CIBIS II	1999	Bisoprolol	2647	34% relative risk reduction in all-cause mortality
BEST	2001	Bucindolol	2708	No significant difference in mortality between the two groups
CAPRICORN	2001	Carvedilol	1959	23% relative risk reduction in all-cause of mortality
COPERNICUS	2001	Carvedilol	2289	31% relative risk reduction in all-cause of mortality
COMET	2003	Metoprolol	2309	17% relative risk reduction in all-cause of mortality
MERIT-HF	1999	Metoprolol	3991	34% relative risk reduction in all-cause of mortality
SENIORS	2005	Nebivolol	2128	No significant difference in mortality between the two groups

**Table 2:** Demographic and clinical characteristic of the study population.

Age (Years)	53 + 7.1
Female sex (n/%)	162 (33.5%)
Ischemic cardiomyopathy (n/%)	270 (56.25%)
LVEDV (ml)	240 + 8
LVEF (%)	31 + 4
Median N-terminal pro–B-type natriuretic (pg/mL)	1720 (886–3854)
NYHA I/II/III/IV (n)	3/354/115/8
Hypertension (n/%)	235 (48.9)
Diabetes (n/%)	263 (54.7%)
Diuretics (n/%)	310 (64.5%)
Digitalis (n/%)	88 (18.3%)
Beta-blockers (n/%)	480 (100%)
ACE-Inhibitors/Angiotensin receptor blockers (n)	400 (83.3%)
Mineralocorticoid antagonist (n)	255 (53.1%)

**Table 3:** Demographic and clinical characteristic according to β-blockers doses.

	Group 1 (289 pts)	Group 2 (191 opts)	*p*
Age (years)	52.3 + 8.2	54 + 6.3	0.67
Female sex (n/%)	87 (30%)	75 (39%)	0.82
Systolic blood pressure (mmHg)	110 ± 12	115 ±10	0.87
Heart rate (b/min)	60 ± 5	58 ± 7	0.65
Serum creatinine (mg/dl)	1.13 + 0.8	1.11 + 0.6	0.76
Ischemic cardiomyopathy (n/%)	160 (55%)	110 (57%)	0.45
Atrial fibrillation (n/%)	85 (29%)	72 (37%)	0.81
QRS duration (ms)	148 ± 16	151 ± 20	0.46
LVEDV (ml)	250 ± 75	230 ± 93	0.61
LVEF (%)	32 ± 3	29 ± 5	0.34
Complete left bundle branch block	289 (100%)	191 (100%)	
Median N-terminal pro–B-type natriuretic (pg/mL)	1523 (886–3500)	1758(892–3854)	0.27
NYHA I/II/III/IV (n)	2/209/75/3	1/145/40/5	0.69
Hypertension (n/%)	130 (44%)	105 (54%)	0.74
Diabetes (n/%)	175 (60%)	88 (46%)	0.22
Diuretics (n/%)	180 (62%)	130 (68%)	0.46
Digitalis (n/%)	50 (17%)	38 (19%)	0.68
Beta-blockers (n/%)	289 (100%)	191 (100%)	0.47
ACE-Inhibitors/Angiotensin receptor blockers (n)	250 (86%)	150 (78%)	0.31
Direct oral anticoagulant (n/%)	85 (29%)	72 (37%)	0.81

**Table 4:** β-blockers doses of the two study groups before and after cardiac resynchronization therapy.

**Group 1**	**Beta-Blockers**	**Dose before CRT**	**Dose after CRT**	***p* Value**
	Bisoprolol (175 pts)	5 ± 1.25 mg	5 ± 2.5 mg	0.34
	Carvedilol (114 pts)	25 ± 18.75 mg	25 ± 18.75 mg	0.21
**Group 2**	**Beta-blockers**	**Dose before CRT**	**Dose after CRT**	
	Bisoprolol (110 pts)	7.5 ± 1.25 mg	11.25 ± 1.25 mg	0.01
	Carvedilol (81 pts)	50 ± 12.5 mg	75 ± 25 mg	0.05

**Table 5:** Clinical and echocardiographic features of the two study groups before and after cardiac resynchronization therapy.

**Group 1**	**Before CRT**	**After CRT**	***p* Value**
LVEDV (mL)	250 ± 75	220 ± 45	0.25
LVEF (%)	32 ± 3	35 ± 3	0.18
NYHA I/II/III/IV	2/209/75/3	4/260/24/0	0.04
Heart rate (b/min)	60 ± 5	60 ± 3	0.55
Blood pressure (mmHg)	100 ± 8	104 ± 7	0.03
**Group 2**			
LVEDV (mL)	230 ± 93	205 ± 72	0.12
LVEF (%)	29±5	33 ± 6	0.09
NYHA I/II/III/IV	1/145/40/5	10/175/5/1	0.02
Heart rate (b/min)	58 ± 7	55 ± 5	0.38
Blood pressure (mmHg)	97 ± 6	103 ± 4	0.02
